# Human Blood Autoantibodies in the Detection of Colorectal Cancer

**DOI:** 10.1371/journal.pone.0156971

**Published:** 2016-07-06

**Authors:** Ola H. Negm, Mohamed R. Hamed, Robert E. Schoen, Richard L. Whelan, Robert J. Steele, John Scholefield, Elizabeth M. Dilnot, H. M. C. Shantha Kumara, John F. R. Robertson, Herbert F. Sewell

**Affiliations:** 1 Immunology, School of Life Sciences, University of Nottingham, Nottingham, United Kingdom; 2 Medical Microbiology and Immunology Department, Faculty of Medicine, Mansoura University, Mansoura, Egypt; 3 School of Medicine, University of Nottingham, Derby, United Kingdom; 4 University of Pittsburgh, School of Medicine, Pittsburgh, United States of America; 5 Mount Sinai Roosevelt, Division of Colon and Rectal Surgery, Department of Surgery, New York, United States of America; 6 Medical Research Institute, Ninewells Hospital and Medical School, Dundee, United Kingdom; 7 Nottingham Digestive Diseases Centre, Nottingham University Hospital, Nottingham, United Kingdom; CCAC, UNITED STATES

## Abstract

Colorectal cancer (CRC) is the second most common malignancy in the western world. Early detection and diagnosis of all cancer types is vital to improved prognosis by enabling early treatment when tumours should be both resectable and curable. Sera from 3 different cohorts; 42 sera (21 CRC and 21 matched controls) from New York, USA, 200 sera from Pittsburgh, USA (100 CRC and 100 controls) and 20 sera from Dundee, UK (10 CRC and 10 controls) were tested against a panel of multiple tumour-associated antigens (TAAs) using an optimised multiplex microarray system. TAA specific IgG responses were interpolated against the internal IgG standard curve for each sample. Individual TAA specific responses were examined in each cohort to determine cutoffs for a robust initial scoring method to establish sensitivity and specificity. Sensitivity and specificity of combinations of TAAs provided good discrimination between cancer-positive and normal serum. The overall sensitivity and specificity of the sample sets tested against a panel of 32 TAAs were 61.1% and 80.9% respectively for 6 antigens; p53, AFP, K RAS, Annexin, RAF1 and NY-CO16. Furthermore, the observed sensitivity in Pittsburgh sample set in different clinical stages of CRC; stage I (n = 19), stage II (n = 40), stage III (n = 34) and stage IV (n = 6) was similar (73.6%, 75.0%, 73.5% and 83.3%, respectively), with similar levels of sensitivity for right and left sided CRC. We identified an antigen panel of sufficient sensitivity and specificity for early detection of CRC, based upon serum profiling of autoantibody response using a robust multiplex antigen microarray technology. This opens the possibility of a blood test for screening and detection of early colorectal cancer. However this panel will require further validation studies before they can be proposed for clinical practice.

## Introduction

Colorectal cancer (CRC) is the second most common malignancy in the western world in terms of incidence and cancer-related mortality and carries a significant economic burden [1, 2]. Prognosis from CRC is convincingly dependent on stage at diagnosis as the most important survival determinant. Thus, early detection and diagnosis is vital to improved prognosis and has great potential to reduce the burden of this disease.

There is good evidence that screening reduces CRC incidence and mortality [3–7]. However, despite the reliable detection of CRC and its precursors by colonoscopy and, in the distal colon and rectum by sigmoidoscopy, the use of these invasive procedures for screening is limited by available resources, costs and low compliance [5, 6, 8]. The established non-invasive tests, such as the guaiac faecal occult blood tests (gFOBT), suffer from low sensitivity. Randomized, controlled trials have shown that annual or biennial fecal occult blood tests (FOBTs) are associated with a 15% to 33% decrease in CRC mortality rates following detection and appropriate intervention [7, 9–11]. However, this impact is limited by low patient acceptability of FOBT and low sensitivity and resulting in high interval cancer rates [12, 13]. Increased rates of completion of screening in an integrated care delivery system were achieved by adoption of the faecal immunochemical test (FIT) [14] and FIT can provide increased sensitivity compared to gFOBT [15]. However, blood tests are likely to be more acceptable than stool tests in population-based screening [16].

Therefore, a cost-effective relatively non-invasive blood test, reliably identifying early CRC and the most advanced precursors, and thereby identifying for colonoscopy those who will most likely benefit, would be extremely desirable but has yet to be developed. In recent years, an increasing number of studies has shown that cancer patients produce detectable autoantibodies (AAbs) against certain tumour associated antigens (TAAs) early during tumour development [17–20], which makes autoantibody signature a particularly promising approach for screening and early detection of CRC even at very early stages of cancer development. Many studies have indicated that no single marker can completely distinguish the cancer group from the healthy controls. However, the combination of multiple markers may provide a promising approach for early detection of cancer [21].

The aim of this study was to evaluate the application of a multiplex antigen microarray utilising a small panel of TAAs to distinguish CRC from controls based upon serum profiling of autoantibody response.

## Material and Methods

### Serum samples

A total of 262 confirmed CRC and normal sample sera were provided from three different cohorts as follows: 100 CRC [53 males and 47 females with a median age of 64.3 (35.5–91.3)] and 100 control samples [36 males and 64 females with a median age of 54.3 (20.5–81.2)] from Pittsburgh, USA, 21 CRC [9 males and 12 females with a median age of 58 (33–89)] and 21 control [7 males and 14 females with a median age of 61 (36–74)] samples from New York, USA and 10 CRC [5 males and 5 females with a median age of 74.9 (57.9–88.5)] and 10 control samples [4 males and 6 females with a median age of 64.6 (51.4–73.2)] from Dundee, UK. Additional controls included samples from 21 patients with autoimmune disorders [7 males and 14 females with a median age of 56.7 (19.3–80)], 9 patients with inflammatory bowel disease (IBD) [4 males and 5 females with a median age of 32.5 (21–83)] and 10 patients with allergy. IgG was purified using Melon^TM^ Gel Spin Plate Kit for IgG Screening (Thermo Scientific), according to the manufacturer’s instructions and stored at -20°C. The serum samples from NY and Dundee were tested in an unblinded manner to demonstrate initially that the test provided cancer/normal discrimination. The largest blood sample set (from Pittsburgh) was tested in a blinded manner to aceess not only cancer/normal discrimination but to look at other features such as the sensitivity and specificity by stage and site. Blood samples were collected at each centre under ethical approval by the relevant ethics committee for that centre. Samples were collected with written consent under a protocol approved by the University of Pittsburgh Institutional Review Board (IRB0411047), the Institutional Review Board of St. Luke's-Roosevelt Hospital Center (IRB 09–113) and and NHS Tayside Ethics Committee. Links providing more detail of protocols and processes involved are as follows: https://edrn.nci.nih.gov/about-edrn/edrn_manualofoperations_4.0.pdf, http://www.colorectalsurgerynyc.com/research/lab-overview.aspx, http://www.tissuebank.dundee.ac.uk/?page=main.

### Tumour associated antigens

#### Tumour-associated antigen selection

A panel of 32 TAAs (non-glycosylated recombinant proteins expressed in *E*.*coli*) were included: P53, SOX2, NY-ESO-1, GBU, MAGE A4, HuD, AFP, Gankyrin, GRP78, HCC1, HDGF, H-Ras1, IMP, p62, RalA, MUC1, CEA, Annexin A1, rhUteroglobulin (CCSP1), K-Ras, APC1, APC2 blocking peptide, SDCCAG8 (NY-CO-8), TDRD6 (NY-CO-45), vWFA2 (CCSP2), ErbB2, RAF1, SCGB1A1, CA19-9, UTP14A (NY-CO-16), K-RAS-Q61H and APC-N. Arrays contained quadruplicates of the chosen TAAs (in native conditions), and included 4 antigens of common pathogens as control targets and a duplicated, 12 point human IgG serial dilution from (50 ug/ml to 24.4 ng/ml).

#### Buffer exchange and pre-array assessment of TAAs

Desalting and buffer exchange of all antigens was performed using Micro Spin columns (PD Mini Trap-G25, GE Healthcare) according to the manufacturer’s protocol. The quality of the antigens was assessed by Western blot and Silver stain (Fluka). Briefly, following mixing in an equal volume of 2X Laemmli sample buffer and heating at 90°C for 5min, similar quantities of TAAs (1 ug) were separated on Novex 4–20% Tris glycine polyacrylamide gels (Invitrogen Life Technologies, USA). After transfer onto nitrocellulose, antigens were detected by labelling with rabbit anti-His antibodies (1:1,000) followed by a horseradish peroxidase (HRP)-conjugated anti-rabbit secondary antibody (1:2,000). Blocking and incubations with antibodies were performed with 0.5% milk in Tris-buffered saline, whereas 0.05% Tween-20 in Tris-buffered saline was used for washing. The Amersham ECL Prime Western Blotting Detection Reagent was used to reveal HRP activity according to the manufacturer’s instructions.

### Microarray protocol

Samples were tested against a panel of TAAs using multiplex microarray system (MMA). Each antigen [100μg/ml in PBS-Tween-Trehalose (50mM)] was loaded onto a 384 well plate (Genetix, UK), and printed in quadriplicate in a 16x16 array format onto aminosialine-coated glass slides (Shott, Germeny) using a Biorobotics Microgrid II arrayer (Microgrid) and a silicon contact pin (Parallel Synthesis Technologies, USA). The serum reactivity was tested against TAAs under native conditions. During printing, the array chamber was set at 20°C. Printed slides were left on the arrayer overnight and processed the next day.

Slides were blocked with 5% PBST-BSA (Sigma) for one hour and washed three times with 0.05% PBS-Tween wash buffer. After a one hour incubation with purified IgG (1:2000 in 5% PBST-BSA) at room temperature with shaking, slides were washed, as above, and 100μl of diluted biotinylated anti-human IgG antibodies (Vector Laboratories; 1:20,000 in antibody diluent) was added to each block for 1 hour. Following another wash, 100μl of 1:2000 diluted streptavidin-HRP (Bio-Rad, USA) was added to each block and slides stored in the dark for 15 minutes. After further washing, 100μl of Bio-Rad amplification reagent was added to each block and slides stored in the dark for 10 minutes. The slides were washed three times with 20% PBS Tween-DMSO and three times with wash buffer. Following this, 100μl of 1:1000 diluted streptavidin-conjugated Cy5 (E-Biosciences, UK) was added to each block and the slides stored in the dark for 15 minutes. After a final wash, slides were rinsed in ultra-pure water and dried by centrifugation. Slides were scanned at 635nm and the data analysed using the GenePix Pro Software (Axon GenePix®). Briefly, the mean background was subtracted from the median fluorescence of each spot and the corrected fluorescence was used to calculate the average fluorescence signal as well as the standard deviation.

### Data analysis and statistics

Arrays were analysed using an existing pipeline for data acquisition and feature filtering for quality control. Data was acquired using an Axon 4200 AL multispectral laser scanner and Axon Genepix Pro software. Poor quality features were flagged for removal based upon mathematical and statistical data addressing spot morphology (circularity and size), signal uniformity (median signal intensity compared to mean signal level), local background and signal to noise ratio. Replicate spots were then processed though customised modules in the R statistical language to general mean signal levels. Interpolation to the IgG standard curve was performed in Graphpad Prism. Signal arithmetic mean and SD, median, range and percentiles for all antigens were all generated during this core analysis. The data for each antigen was assessed both via a visual plot of cancer versus controls and also by assessing the sensitivity and specificity for CRC using an optimized cut-off in the control population.

## Results

### Pre-array assessment of TAAs

**Fig 1:**
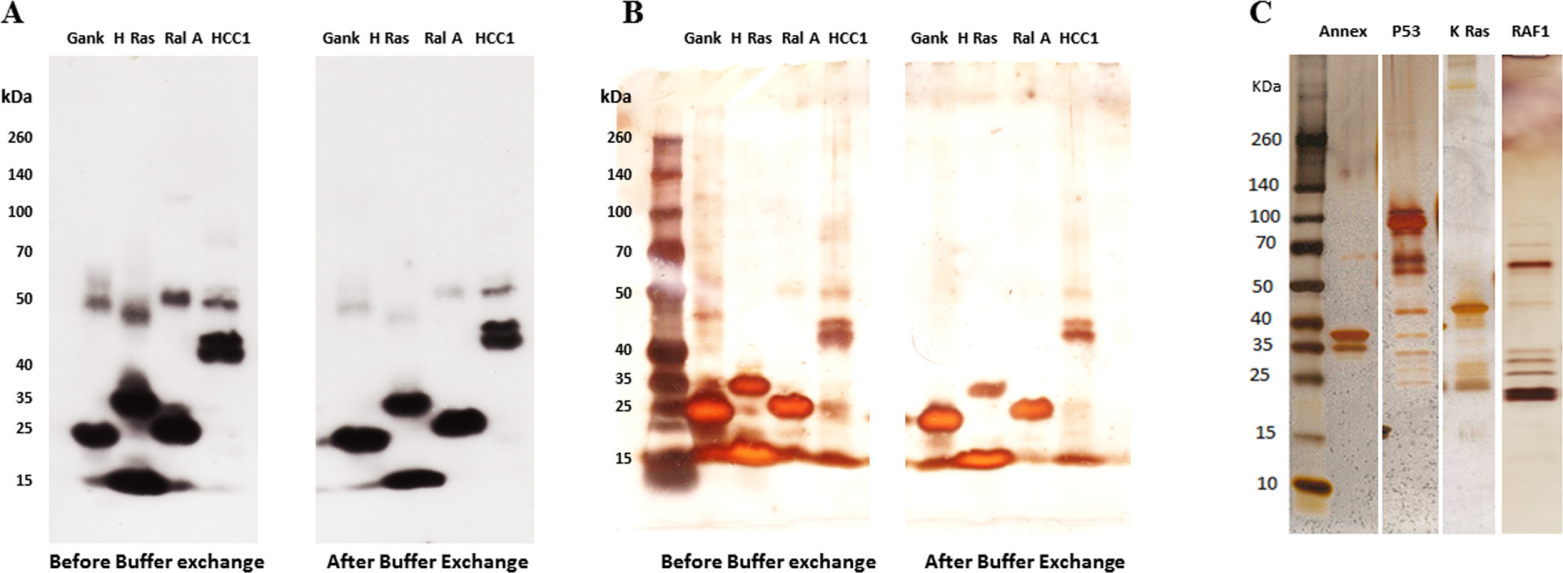
A representative analysis of a selection of TAAs by Western blot (WB) and Silver stain. Samples were run on 4–20% precast polyacrylamide gels with a broad range protein markers. The 4 antigens (Gankyrin, H-Ras, RalA, and HCC1) were then either probed with anti-His antibody (**A**) or silver stained (**B**). Silver stain was used to check the purity of the antigens. The expected molecular weight of the examined antigens, Gankyrin, H-Ras, RalA, and HCC1 are 25, 40, 22 and 64 kDa respectively. The similar bands obtained before and after buffer exchange by both WB and silver stain indicate that the buffer exchange technique had no effect on the integrity of the antigens. **C**: Silver stain of 4 TAAs: Annexin, P53, KRAS and RAF1 (the expected molecular weight are 39, 53, 57 and 73 kDa respectively).

All TAAs obtained were assessed prior to their application to microarrays. Antigens were tested for antigen concentration, and analysed upon receipt, by SDS-PAGE gel electrophoresis, western blotting with specific anti-tag antibodies (Fig 1A) and silver staining (Fig 1B and 1C) to determine purity and integrity. This process was reapplied after buffer exchange to ensure protein integrity and concentration was not compromised by the process (Fig 1B).

### Technical development

**Fig 2:**
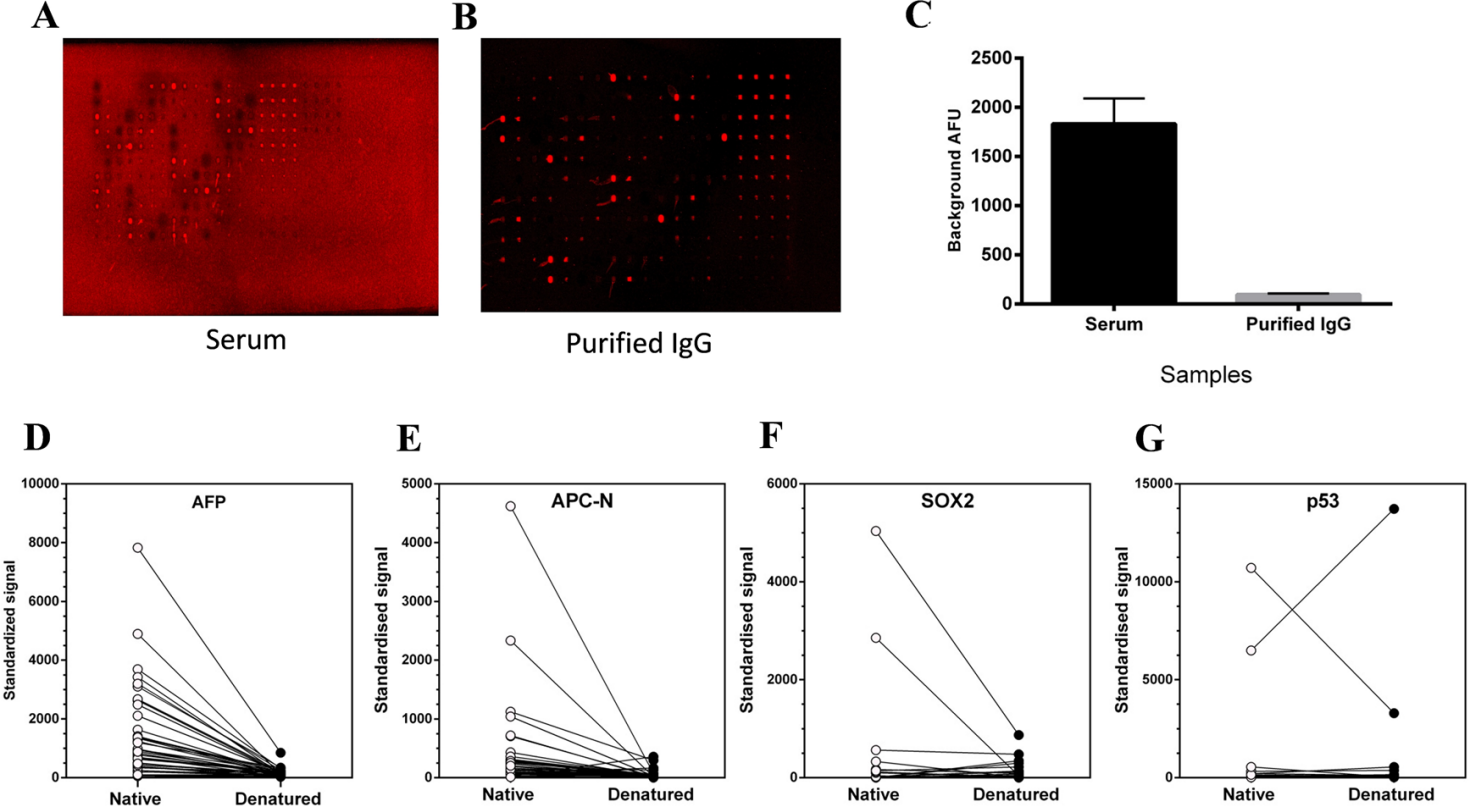
Technical development in microarray. **A-C**: Demonstration of improvements in array background for a serum sample with high background signal when purified IgG (**B**) is used in place of whole serum (**A**). These arrays were processed at the same time and scanned under the same conditions. (**C**) displays the average background measurements taken from empty spots in both slides when the whole serum was used and when the purified IgG was used (n = 35). D-G: Effects of TAA denaturation of serum antibody binding. Representative antigens (**D**: AFP, **E**: APCN, **F**: SOX2 and **G**: P53) were printed as native protein or after heat-denaturation in the presence of 20mMDTT. Serum samples (n = 42) from a CRC cohort were used to examine alterations in autoantibody reactivity between the native and denatured TAAs. The mean of the signal from each TAA (4 replicates) is presented (n = 4).

Sera were assessed at multiple dilutions in our proprietary dilution buffer to determine a blanket optimum dilution which provided low background for the array surface in use. In addition, a subset of sera was purified by a one-step method to yield the IgG fraction alone, and a second fraction, devoid of IgG (ie containing IgM, A, E and other serum components). These were examined on TAA arrays to compare background and foreground signal relative to complete serum. Fig 2A–2C shows an example of arrays probed with a serum sample with high background signal, before and after IgG purification. The improvement in array background is both visible (comparing A with B) and measureable (2C).

Antigens were examined under native and reducing conditions for serum reactivity to the TAAs. Fig 2D–2G shows representative TAAs and a predominant reduction in AAb signal across a spectrum of CRC sera (42 in total) when the TAAs are denatured. Fig 2G reveals one serum showing exception to this trend, however in this case the signal is extremely high whether the TAA is native or denatured. In all further analyses the TAAs were printed onto arrays in native form.

**Fig 3:**
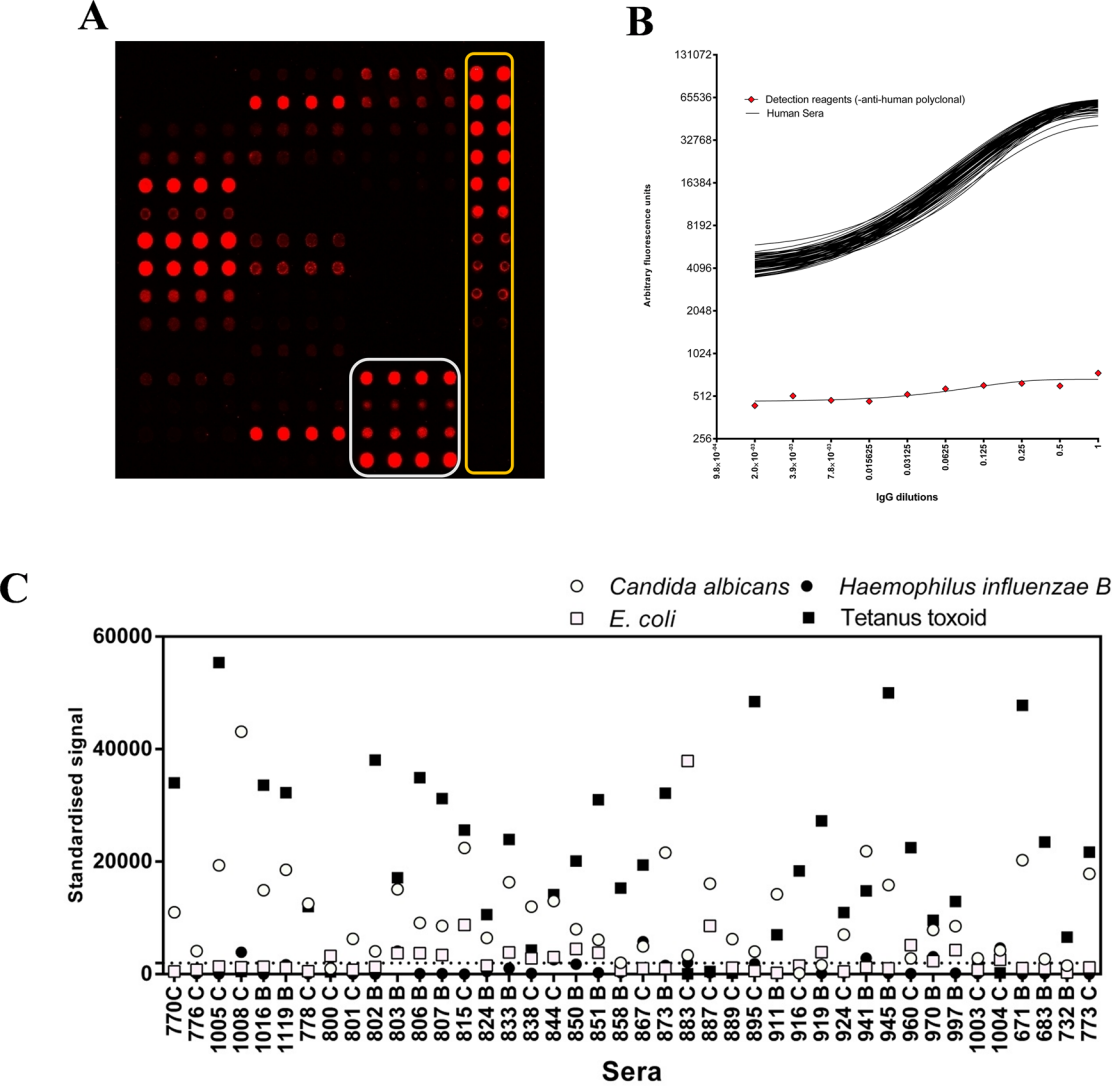
Internal quality control measures on MMA. **A:** representative array image showing layout and inclusion of printed Human IgG standard curve (outlined in yellow) and 4 control antigens each as quadruplicate features, printed horizontally (outlined in white). The Human IgG standards are used to normalise all TAA features and arrays to a common scale. **B:** Overlay of 72 individual standard curves from 72 arrays probed with patient serum and one array probed with all detection reagents, except the anti-human polyclonal antibody (red diamonds). The dynamic range of the assay is shown in log2 scaled y axis. X axis represents the dilution of IgG (starting at 50ug/ml). **C**: Normalised signals to 4 control antigens obtained from 42 serum samples applied to optimised TAA arrays. Strong responses are seen to tetanus toxoid and *Candida albicans* antigen preparations.

Internal QC measures on each array were devised to support inter-array normalisation, assay performance and data acquisition machine performance monitoring. These measures included the addition of a replicated serial dilution of purified human IgG to verify function of the detection system and provide a standard curve of human IgG against which AAb responses could be calibrated (Fig 3A and 3B). Antigens from 4 known human pathogens, where the majority of normal individuals would be expected to have some existing IgG protective antibody response were incorporated onto each array. These were used as an indirect measure of serum quality and antibody reactivity. These preparations included an *E*. *coli* lysate, tetanus toxoid and *Haemophilus influenzae B* (from NIBSC) and a *Candida albicans* mixed antigen preparation (Fig 3A). These control antigen signals were examined for each array as an indicator of sample integrity. Fig 3C shows a plot of the responses seen in 42 samples for each of the 4 control antigens. *Candida albicans* and tetanus toxoid are seen as generally good targets, whilst *E*. *coli* and *H*. *influenzae B* antigen did not yield highly positive results from the majority of serum samples.

### Cohort results associated with TAA responses

**Fig 4:**
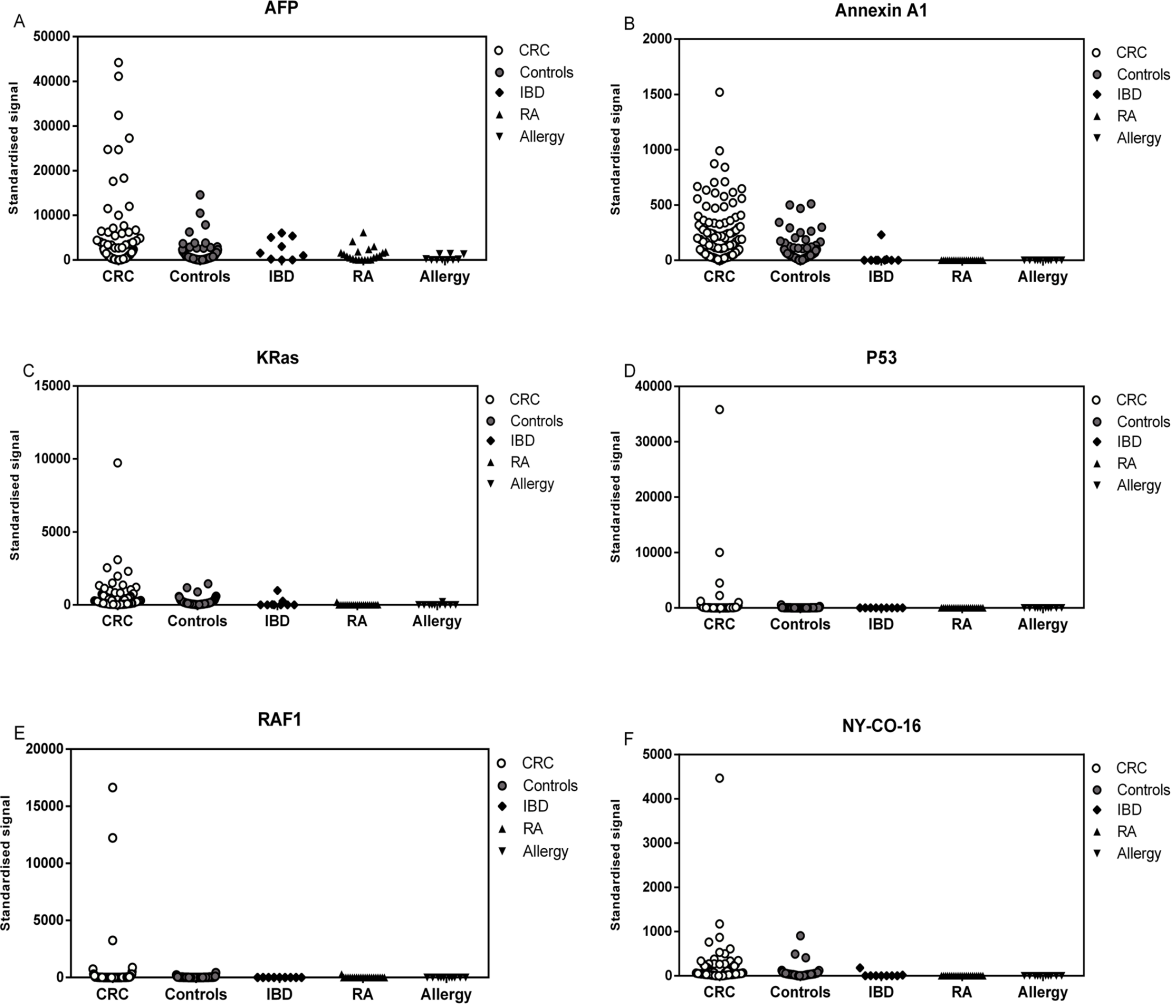
Representative antigen panels selected for all sample sets. The figure shows the autoantibody responses of the CRC patients (n = 131), healthy controls (n = 131), autoimmune (n = 21), IBD (n = 9) and allergy (n = 10) cohorts to a panel of TAA(s): data shows reactivity to AFP (A), Annexin (B), KRas (C), P53 (D), RAF1 (E) and NY-CO16 (F).

After the assay development and optimisation steps were complete, all samples were processed through the system. Arrays contained quadruplicates of all 32 TAAs chosen for this study, and included 4 common pathogens as control targets and a duplicated, 12 point human IgG serial dilution from (50 ug/ml to 24.4 ng/ml). The IgG dilution series was used to interpolate the equivalent IgG levels bound to each TAA and control feature on the array. Sera (purified IgG) from 3 different cohorts; 200 sera (100 CRC and 100 controls) from Pittsburgh, 42 sera (21 CRC and 21 matched controls) from New York and 20 sera (10 CRC and 10 controls) from Dundee were tested against a panel of multiple antigens (Fig 4). TAA specific IgG responses were interpolated against the internal IgG standard curve for each sample. Normality testing classified the data for most antigens as non-parametric with the minority of antigens showing normal distribution (S1 Fig). Individual TAA specific responses were examined in each cohort to optimise antigen cut-offs (for maximal cancer: normal differentiation), to establish a robust scoring method to generate sensitivity and specificity for each TAA. The 95^th^ percentile of the median values of the healthy control group for each TAA was calculated and used as a cut-off point.

Sensitivity and specificity of individual TAAs and panels of TAAs were calculated to establish how combinations of this test set of TAAs would provide good discrimination between cancer-positive and normal serum samples. S3 Fig demonstrates sensitivity and specificity in the Pittsburgh, New York (NY) and Dundee samples for the same panel of 6 tumour antigens (p53, AFP, K RAS, Annexin, RAF1 and NY-CO16) based upon the same cut-off point (95^th^ percentile of the healthy control group). Pittsburgh samples have a sensitivity and specificity of 75% and 78% respectively while the figures for NY are 43% and 81% respectively and for Dundee are 40% and 100% respectively.

In the largest dataset (Pittsburgh) we were able to look at the relative value of individual TAAs compared to the panel. The panel of 6 TAAs gave a sensitivity and specificity of 75% and 78% respectively. The sensitivity and specificity of individual TAAs are shown in Table 1. This shows clearly the added value of a panel of AAbs compared to the results of a single TAA. This is similar to data on lung cancer where again a panel of AAbs was better than any single one.

**Table 1:** The relative value of individual TAAs compared to a panel of 6 TAAs in the largest dataset (Pittsburgh).

Antigen/Panel	Sensitivity (%)	Specificity (%)
AFP	27	95
P53	26	95
K Ras	27	96
NY-CO-16	41	95
RAF1	18	95
Annexin	29	94
A panel of the 6 TAAs	75	78

Cut-off cofactor applied was based upon the 95th percentile of the healthy controls.

**Fig 5:**
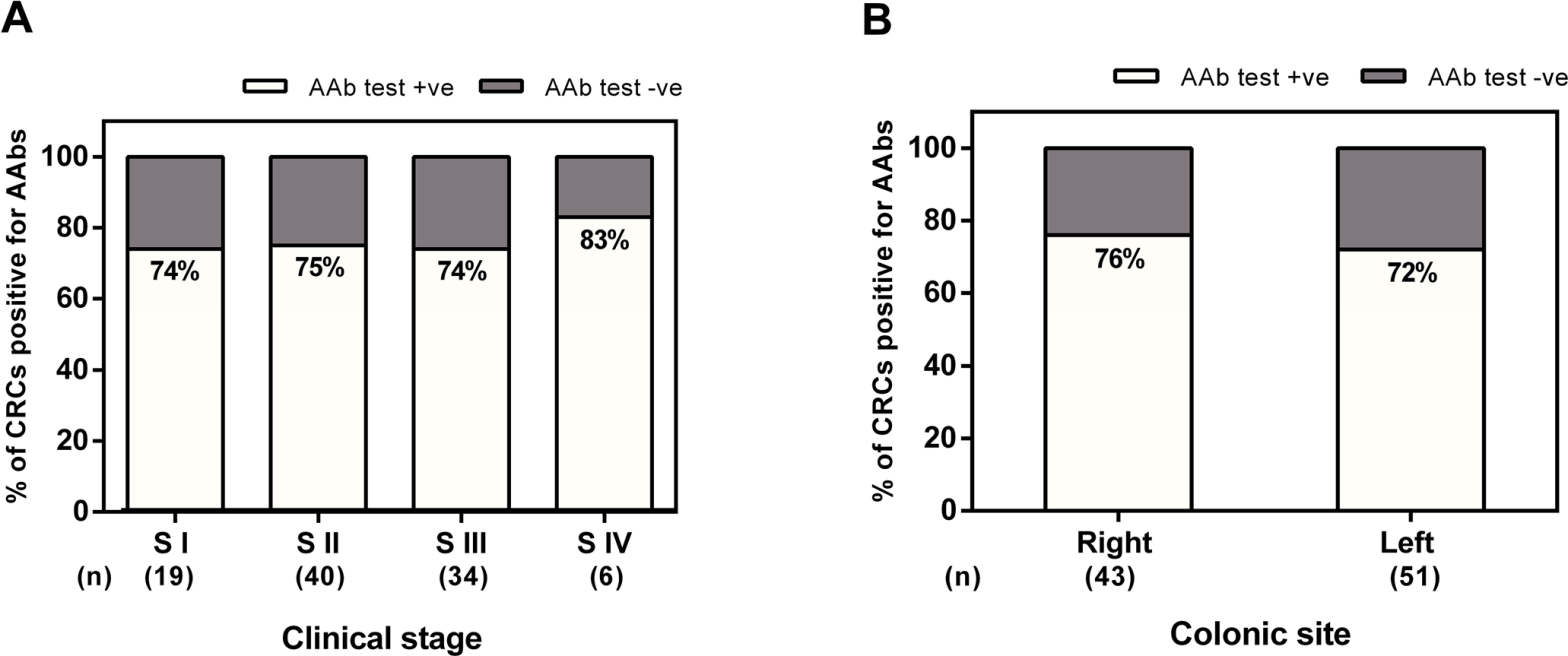
**Sensitivity of of the Pittsburgh CRC sample set (n = 100) by stage (A) and site (B).** A: The percentage of CRC patients positive for AAb test (i.e sensitivity) by stage of disease at presentation for the Pittsburgh CRC samples. Cut-off point = 95th percentile of the control group. B: The percentage of CRC patients positive for AAb test (i.e sensitivity) by the site (left or right side of the colon).

We also looked in the Pittsburgh dataset at the important correlations of AAbs versus stage of disease and CRC location. This was not possible in the other two datasets due to the smaller sample sizes. Fig 5A indicates that the percentage of positivity of stage I (S I) is similar to that stage II (S II), stage III (S III) and stage IV (S IV). The data in Fig 5A shows that the percentage of positivity of S I, S II, S III, and S IV (73.6%, 75.0%, 73.5% and 83.3, respectively), which indicate that the sensitivity is not stage dependent. The data also show no difference in AAb detection by CRC location (76% sensitivity for left side CRCs and 72% for right side CRCs) (Fig 5B) or gender (81% sensitivity for males and 67% for females).

Sera from patients with autoimmune diseases (n = 21), inflammatory bowel disorders (n = 9) and allergies (n = 10) were tested along with known AAb positive CRC (n = 20) and healthy controls (n = 18) against a subpanel of TAAs. The autoimmune/IBD/Allergy groups (except for one IBD sample) showed lower values than the healthy controls (Fig 4).

## Discussion

This study demonstrates the initial development of a robust, non-invasive blood test for detection of CRC with high sensitivity and specificity using a panel of selected TAAs; such a test should have a high patient acceptability rate. Our route has been to convert a commonly used platform i.e. 96-well, microtitre-based ELISA to solid-phase protein microarrays, which offer numerous advantages including: a much increased capacity for multiplexing detection of a range of specific AAbs; vastly reduced requirements for TAAs, serum and reagents; increased assay robustness; and the potential for much greater throughput than that achievable by ELISA-based assays.

Multiple technical enhancements have been applied and verified. These have enhanced various assay parameters to dramatically improve the capabilities of the system, over and above those offered by conventional ELISA-based TAA detection and quantification methods, such as that used in the Lung-EDT approach [22]. Optimizations included: i) antigen sources and purity; ii) optimizing TAA printing uniformity and antigenic epitope retention; iii) blocking and buffer choices to provide optimal signal to noise ratios; iv) internal QC measures to support inter-array normalization, assay performance and data acquisition performance monitoring; v) application of an existing pipeline (in R) for data acquisition and quality control to this early cancer detection project.

The body of published studies [reviewed in [21]] and a small pilot study on AABs detected in a small number of CRC samples previously run by our group provided the basis for the initial candidate TAAs (n = 32) selected for this study. Although, most of the examined autoantibody markers had relatively low individual sensitivity (<25%), sensitivity and specificity of combinations of TAAs provided good discrimination between cancer positive and normal serum. Using a 95th percentile cut-off the sensitivity and specificity of New York sample set tested against a panel of 6 antigens; p53, AFP, K RAS, Annexin, RAF1 and NY-CO16 were 43% and 81%, respectively and Pittsburgh sample set tested against the same panel were 75% and 78%, respectively. The achieved sensitivity and specificity were similar for both sample sets, indicating robustness of the microarray format across different centres. However, one impact of small numbers in sample sets is that the panel of TAAs which detect most cancers in one sample set might not be the same as in another. Previous studies showed evident discrepancy in the the diagnostic performance of different autoantibody markers which may be attributed to different definition of the cut-off value, diverse detection methods (e.g., ELISA, protein array), diverse study designs, study population, and sample size [21, 23]. Thus combination of multiple markers is required to enhance sensitivity for autoantibody detection and diagnostic performance cancer screening. Babel et al., 2009 [24] applied a protein microarray to simultaneously screen 8000 proteins and identified a combination of three autoantibody markers (PIM1, MAPKAPK3 and ACVR2B) that yielded a high diagnostic efficacy, with 84% sensitivity and 71% specificity.

Besides the ability to distinguish between cancer and normal populations, identifying early stage disease was a further avenue which was explored. The observed sensitivity in Pittsburgh sample set in different clinical stages; stage I, stage II, stage III and stage IV was similar (73.6%, 75.0%, 73.5% and 83.3%, respectively), which indicates that the sensitivity is stage independent. This similar sensitivity for early as for late stages of CRC is quite a significant feature as the survival of patients diagnosed with the earliest stage of disease is over 90% compared to only 6.6% of those diagnosed with advanced disease [25]. Furthermore, the reactivity of sera from non-malignant immune system activation controls (e.g. autoimmune diseases, inflammatory bowel disorders and allergies) indicated that inflammatory disorders and non-malignant immune system activation do not appear to adversely or non-specifically affect the performance of the blood test for early detection of CRC using this modest sized sample set.

For a blood test to be used for screening purposes (ie a first line test) it should be able to discriminate high risk groups (eg adenomatous polyps, advanced adenomas) and early stage colorectal cancers from the general population (average risk group).We have preliminary evidence (unpublished) that our blood test detects a significant proportion of cases of adenomatous polyps. Together with the current findings reported herein (detection of Stage 1-IV CRC) indicates that this blood test has the potential to be used in first line screening protocols thus enabling the more targeted use of colonoscopy. Such a blood test with the desired characteristics may complement the use of FIT for triaging cases (in risk prediction algorithms for cancer prevention and screening) with improved specificity for colonoscopy referral. Indeed future head to head studies of the blood test versus FIT should define the performance of the blood test in comparison to the stool test.

Randomised trials, using gFOBT followed by colonoscopy, have shown early detection with appropriate intervention results in mortality reduction of 16% [9, 26]. While significant, the impact is limited by a number of factors–e.g majority of patients still present with late disease, low patient acceptability of gFOBT (Uptake: UK 57%; European 34%) and the sensitivity (50%) and specificity (75%) for gFOBT are low. It is noteworthy that recent reported studies using quantitative FIT indicates improved performance characteristics with sensitivity of ~75% (but only 25% for advanced adenomas) and specificity similar to gFOBT [25]. Although the mortality reduction for one off and repeat sigmoidoscopy appears higher (22%-31%) [5, 6, 8], patient acceptability remains low (45%-58%) with associated health inequalities (most affluent quintile uptake rate (63%) and most deprived quintile (38%) [27]. Furthermore the effect is limited to distal CRC. Uptake of one off colonoscopy alone is even lower (24.6%) and has significant cost implications. The better acceptance of a blood test is supported by a study of patients who refused colonoscopy screening: 106 of the 109 subjects accepted an alternative non-invasive method (97%); 90 selected the Sept9 blood test (83%) and 16 selected a stool test (15%) [16]. An autoantibody blood test would have greater patient acceptability as blood tests are accepted across the population and therefore would lead to greater health equality. Given the results in this population who refused colonoscopy, it is likely that with a patient compliance rate approaching 90% (i.e. combination of patients who declined colonoscopy but accepted either Sept9 blood test or FOB [16], a test specificity of 80.9% and sensitivity of 61.1% this would result in detection of 55% of the total number of CRCs with a recall of 17% of the normal population–more than a doubling of the total number of CRCs currently detected. Several recent studies have investigated blood circulating miRNAs such as miR-92, miR-21, miR-409-3p, miR-7, and miR-93 and have reported their potential clinical relevance as a non-invasive biomarker for the diagnosis of CRC patients [28–31].

The first autoantibody ELISA-based test for early lung cancer detection (EarlyCDT-Lung) came from our group. It is available in the USA and is being assessed by the National Health Service (NHS-Scotland, UK) in a RCT of 10,000 high risk individuals [19, 22, 32, 33]. A recent report on the results of this study after more than 9,000 participants confirmed the specificity to be 91%, as predicted, with > 41% sensitivity which are consistent with our previous publications. Furthermore the majority of the cancers in the EarlyCDT-Lung positive group which had been staged were early stagetumours [34]. This together with our pilot work on CRC, indicates the autoantibody approach could become a platform technology for early detection of a wide range of solid tumours. The microarray allows inclusion of a large number of TAAs and ultimately development of a pan-cancer test. Microarray technologies for AAb detection have scalability and economy of sample and reagent use, making them ideal tools for providing cost-effective, comprehensive CRC screening compared to previous published work with ELISA assays, thereby having the potential to significantly improve clinical outcomes by detecting early stage CRC. Our test could also be used in the assessment of symptomatic patients. This could be a valuable tool for both primary and specialty care physicians.

In conclusion, by the application of an optimised multiplex antigen microarray we have shown reproduciblity of results on samples from 3 separate centres with a microarray test. In the largest blood sample set (from Pittsburgh) which gave 78% specificity and 75% sensitivity—sensitivity is not stage dependent (ie AAbs are detected as frequently in early stage I CRC as they are in late stage III/IV). The sensitivity was also similar for right and left sided CRC and with similar detection by stage, site and gender, highlighting thr robustness of the technology and the wide applications of such a blood test. Optimized marker panels with validation in large screening populations are needed.

## Supporting Information

S1 Fig Histograms of the reactivity of healthy controls to a panel of TAAs. The histograms depict the number of values of a specified reactivity of the healthy controls to AFP (A), Annexin A1 (B), P53 (C), KRas (D), RAF1 (E) and NY-CO16 (F). The graphs show asymmetric data sets; right skewed. The data were analysed using the D'Agostino-Pearson omnibus normality test.
(TIF)

S2 Fig A representative antigen panel selected for 3 sample sets. The figure shows reactivity of samples from Pittsburgh (A; n = 100 CRC and 100 controls), New York (B; n = 21 CRC and 21 controls) and Dundee (C: n = 10 CRC and10 controls). Each graph shows side-by-side comparisons of AAb responses to a selection of TAAs (AFP, Annexin A1, GBU, GRP78, HCC1, NY_ESO-1, P53, CCSP1, NY-CO-8, SOX2, NY-CO-45, NY-CO-16 and CCSP2) for clinically confirmed CRC (white circles) and control (grey circles).
(TIF)

S3 Fig Sensitivity and specificity for the New York and Pittsburgh sets using different cut-off points. **A:** Sensitivity and specificity for the New York (21 CRC and 21 controls), Pittsburgh (100 CRC and 100 controls) and Dundee (10 CRC and 10 controls) sets against a panel of 6 antigens (p53, AFP, K RAS, Annexin, RAF1 and NY-CO16). Cut-off point = 95th percentile of the control samples. **B:** Sensitivity and specificity for the New York (21 CRC and 21 controls), Pittsburgh (100 CRC and 100 controls) and Dundee (10 CRC and 10 controls) sets against a panel of 6 antigens (p53, AFP, K RAS, Annexin, RAF1 and NY-CO16). Cut-off point = 2 standard deviations (SD) above the mean of the control serum samples, in line with our previous approach for lung cancer [35, 36] and hepatocellular cancer [37].
(TIF)
