# Supplementary figures and images for: Human Blood Autoantibodies in the Detection of Colorectal Cancer
Source: PLoS One. 2016 Jul 6;11(7):e0156971. doi: 10.1371/journal.pone.0156971 (PMC4934916; doi:10.1371/journal.pone.0156971)

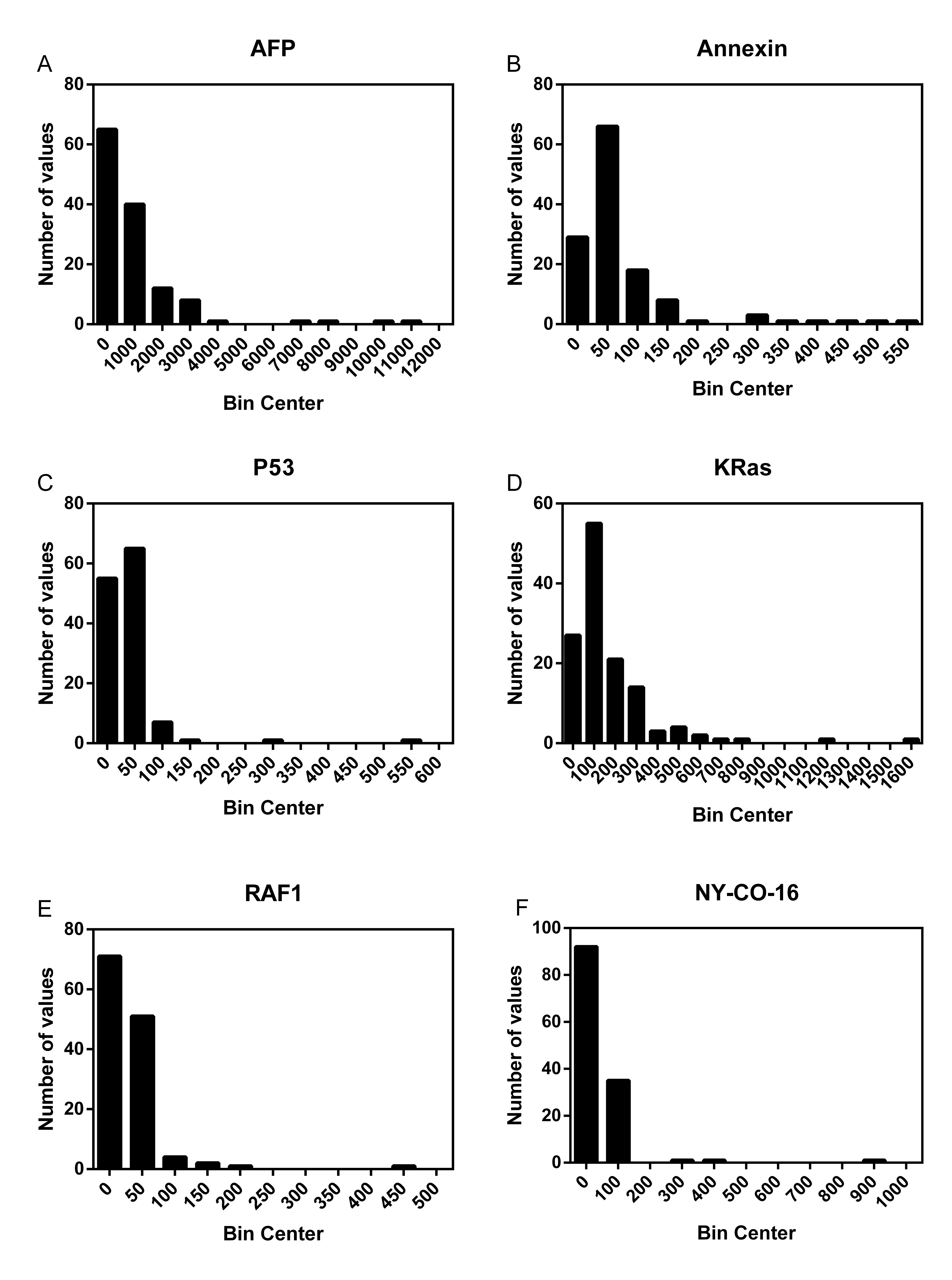

Supplement: S1 Fig — The histograms depict the number of values of a specified reactivity of the healthy controls to AFP (A), Annexin A1 (B), P53 (C), KRas (D), RAF1 (E) and NY-CO16 (F). The graphs show asymmetric data sets; right skewed. The data were analysed using the D'Agostino-Pearson omnibus normality test. (TIF) [file pone.0156971.s001.tif]

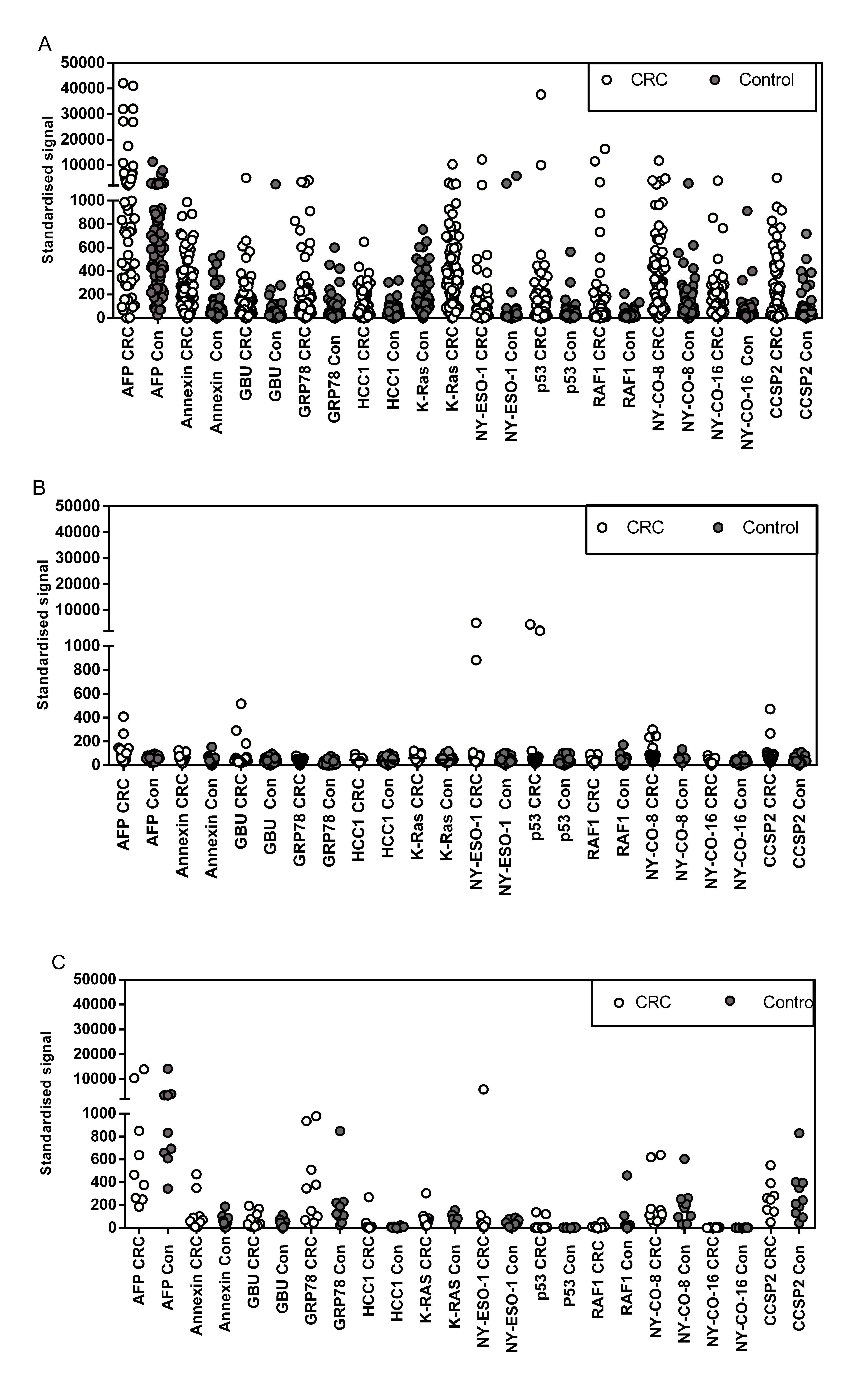

Supplement: S2 Fig — The figure shows reactivity of samples from Pittsburgh (A; n = 100 CRC and 100 controls), New York (B; n = 21 CRC and 21 controls) and Dundee (C: n = 10 CRC and10 controls). Each graph shows side-by-side comparisons of AAb responses to a selection of TAAs (AFP, Annexin A1, GBU, GRP78, HCC1, NY_ESO-1, P53, CCSP1, NY-CO-8, SOX2, NY-CO-45, NY-CO-16 and CCSP2) for clinically confirmed CRC (white circles) and control (grey circles). (TIF) [file pone.0156971.s002.tif]

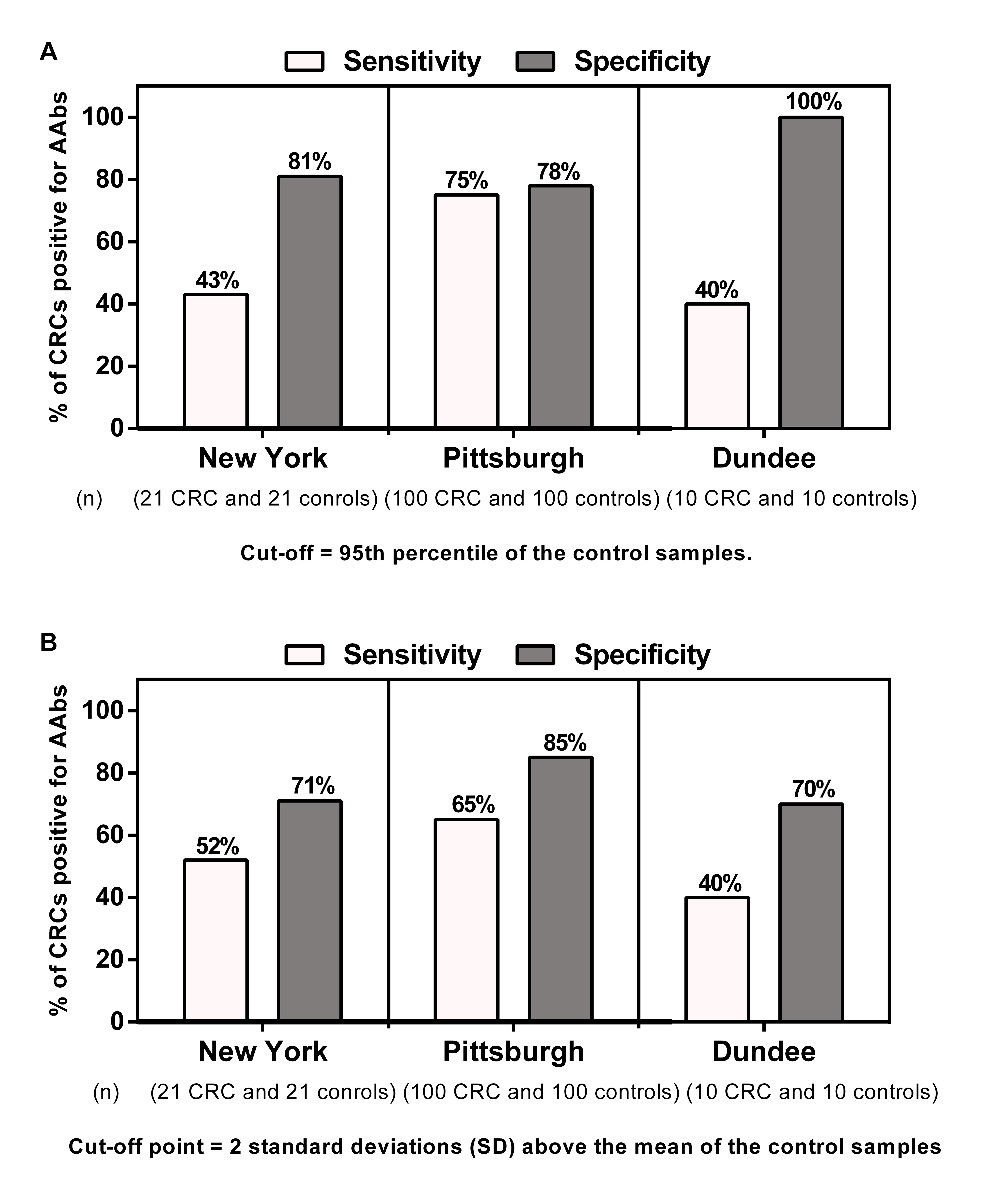

Supplement: S3 Fig — A: Sensitivity and specificity for the New York (21 CRC and 21 controls), Pittsburgh (100 CRC and 100 controls) and Dundee (10 CRC and 10 controls) sets against a panel of 6 antigens (p53, AFP, K RAS, Annexin, RAF1 and NY-CO16). Cut-off point = 95th percentile of the control samples. B: Sensitivity and specificity for the New York (21 CRC and 21 controls), Pittsburgh (100 CRC and 100 controls) and Dundee (10 CRC and 10 controls) sets against a panel of 6 antigens (p53, AFP, K RAS, Annexin, RAF1 and NY-CO16). Cut-off point = 2 standard deviations (SD) above the mean of the control serum samples, in line with our previous approach for lung cancer [35, 36] and hepatocellular cancer [37]. (TIF) [file pone.0156971.s003.tif]
